# Dynamic evolution of NK cells and immune remodeling mediated by CRS + HIPEC: prognostic mechanisms and therapeutic implications for malignant peritoneal mesothelioma

**DOI:** 10.1186/s12957-025-04019-2

**Published:** 2025-11-03

**Authors:** Yi-Tong Liu, Qi-Di Zhao, Xin-Li Liang, Ru Ma, Yan-Dong Su, Rui Yang, Tian Wei, He-Liang Wu, Yu-Bin Fu, Yu-Run Cui, Yang Yu, Bing Li, Yan Li

**Affiliations:** 1 Department of Peritoneal Cancer Surgery, Beijing Shijitan Hospital, Capital Medical University, Beijing, 100038 China; 2 Department of Peritoneal Oncology, Beijing Tsinghua Changgung Hospital, School of Clinical Medicine, Tsinghua Medicine, Tsinghua University, Beijing, 102218 China

**Keywords:** Malignant peritoneal mesothelioma, Natural killer cells, Cytoreductive surgery, Tumor microenvironment

## Abstract

**Background:**

Malignant peritoneal mesothelioma (MPM) is a highly aggressive peritoneal malignancy with a significant recurrence rate following cytoreductive surgery (CRS) combined with hyperthermic intraperitoneal chemotherapy (HIPEC). There is an urgent need to investigate novel therapeutic strategies for MPM. Natural killer (NK) cells exhibit rapid responsiveness in anti-tumor immunity; however, NK cells’ dynamic evolution and clinical significance in MPM remain unclear.

**Methods:**

This study retrospectively enrolled 80 newly diagnosed MPM patients (preoperative group) and 64 patients who underwent CRS + HIPEC (postoperative group). The level of NK cells (CD3^−^CD56^dim^CD16^+^) in peripheral blood was quantified using flow cytometry. Univariate and multivariate regression analyses were performed to evaluate the association between NK cell counts and clinicopathological characteristics, intraoperative events, and prognosis. A multivariate prediction model for NK cell recovery was established.

**Results:**

41 patients (51.3%) exhibited decreased NK cell levels preoperatively, which were significantly associated with an increased risk of thrombosis (*P* = 0.023), intraoperative plasma transfusion (*P* = 0.004), and prolonged hospitalization duration (*P* = 0.023). Postoperative dynamic changes in NK cell levels were found to correlate with Karnofsky performance scale (KPS) scores (*P* = 0.048) and elevated levels of IL-4, IL-5, IL-6, and IL-8 (*P* < 0.05). Multivariate analysis revealed that the volume of intraoperative plasma transfusion was an independent correlated factor for preoperative NK cell reduction (*P* = 0.013), while a low KPS score was an independent predictor of postoperative NK cell decline (*P* = 0.048). Survival analysis indicated that a high perioperative stress score (PSS) (*P* = 0.015), lymph node metastasis (*P* = 0.015), significant intraoperative blood loss (*P* = 0.013), low preoperative CD8⁺ T cell levels (*P* = 0.001), and reduced postoperative IL-17 expression (*P* = 0.013) were independent adverse prognostic factors for overall survival (OS). Furthermore, the dynamic NK cell recovery model demonstrated that baseline NK cell levels, peritoneal cancer index (PCI), CD8⁺ T cell status, and postoperative recovery time all significantly influenced the immune remodeling process (all *P* < 0.001).

**Conclusions:**

Preoperative NK depletion correlated with thrombosis and surgical risks, while postoperative NK recovery was influenced by KPS, specific cytokines (IL-4/5/6/8), and was significantly enhanced after CRS + HIPEC.

**Supplementary Information:**

The online version contains supplementary material available at 10.1186/s12957-025-04019-2.

## Background

Malignant peritoneal mesothelioma (MPM) is a rare malignant tumor originating from peritoneal mesothelial cells, with an annual incidence of approximately 1–2 cases per million [1]. It accounts for 7%−30% of all mesotheliomas [2, 3]. In 2020, the prevalence of MPM in China was 2.6 cases per million, corresponding to a total of 3,737 patients [4]. The Peritoneal Surface Oncology Group International (PSOGI) recommends cytoreductive surgery combined with hyperthermic intraperitoneal chemotherapy (CRS + HIPEC) as the standard treatment regimen [5], which has been shown to increase survival to approximately 3 years [6]. However, despite complete cytoreduction, the recurrence rate remains high [7]. Consequently, identifying novel therapeutic strategies to improve long-term patient outcomes represents an urgent clinical challenge.

Natural killer (NK) cells, as the core effector cells of the innate immune system, exhibit dual anti-tumor effects by directly lysing tumor cells and modulating adaptive immunity [8]. Human peripheral blood (PB) NK cells can be categorized into CD3^−^CD56^dim^CD16^+^ cells (cytotoxic subset, >90%) and CD3^−^CD56^bright^CD16^−^ cells (immunomodulatory subset, < 10%) [9–11]. Their activation does not depend on antigen presentation but rather relies on the “missing self” recognition mechanism to rapidly respond to tumor cells [12, 13]. Recent studies have demonstrated that the tumor microenvironment dynamically regulates NK cell function; high tumor burden suppresses NK cell proliferation through TGF-β secretion [14], whereas immune or targeted therapies can partially restore their cytotoxicity [15]. Although the role of NK cells in peritoneal metastatic cancers, such as gastric cancer, has been preliminarily elucidated [16], the dynamic changes and clinical significance of NK cells in MPM remain unclear. This study is to systematically analyze the dynamic alterations of PB-NK cells of MPM patients before and after surgery, to investigate their correlation with clinicopathological characteristics, treatment response, and prognosis, and to establish a predictive model for NK cell recovery, thereby providing a theoretical foundation for MPM immunotherapy.

## Materials and methods

### Case screening

This study received approval from the Institutional Review Board of Beijing Shijitan Hospital, Capital Medical University (Approval No.: 2015- [28]). All patients provided written informed consent before undergoing CRS + HIPEC. From May 2023 to February 2025, a total of 80 patients newly diagnosed with MPM were retrospectively enrolled in this study. Among them, 64 patients who underwent CRS + HIPEC also had postoperative NK cell activity assessments and were included in the postoperative analysis. All MPM diagnoses were confirmed through retrospective analysis of the hospital database. All included patients met the established inclusion and exclusion criteria for CRS + HIPEC [5], and possessed comprehensive clinicopathological data as well as follow-up information. Venous blood samples were collected after a fasting period of 6–8 h. Following EDTA anticoagulation, the samples were processed and analyzed within one hour to ensure accuracy and reliability.

### NK cell detection

#### Flow cytometry

PB-NK cell subsets (CD3^−^CD56^dim^CD16^+^) were analyzed by flow cytometry using a FACSCanto II instrument and standardized antibodies (CD3: BD Biosciences, catalog number 340662; CD56: BD Biosciences, USA, catalog number 340723). The detection procedure adhered to the immunological counting guidelines published by the Clinical and Laboratory Standards Institute (CLSI) in 2007 [17]. Preoperative NK levels were measured ≤ 7 days before Surgery, postoperative levels at 30± 5 days.

#### Patient grouping

Patients were categorized into three groups based on preoperative NK cell counts: (1) pre-decreased group: < 155 cells/µL; (2) pre-normal group: 155–550 cells/µL; (3) pre-increased group: >550 cells/µL. Thresholds were derived from the 2.5–97.5% interval of healthy donors: [NK% (8.0–26.0%)] × [Lymphocyte count (1100–3200 cells/µL)].

The rate of change was calculated as follows:

1 $$Change\;rate\;=\;\frac{Postoperative\:PB-NK\:cell\:count-\:Preoperative\:PB-NK\:cell\:count}{Preoperative\:PB-NK\:cell\:count}\times100\%$$

Based on the rate of change after surgery, patients were further divided into three groups: (1) post-decrease group: < −10%; (2) post-stable group: −10–10%; (3) post -increase group: >10%. The ± 10% change criterion provides a buffer zone to distinguish biologically meaningful changes from assay variability.

### Research indicators

#### Clinicopathological features

Gender, age, body mass index (BMI), previous treatment history, prior surgery score (PSS), Karnofsky performance status (KPS), histological type (epithelioid or non-epithelioid), lymph node metastasis (yes or no), vascular invasion (yes or no), Ki-67 proliferation index was recorded.

#### CRS + HIPEC-related surgical parameters

Operation duration, peritoneal cancer index (PCI), tumor cytoreduction score (CC), intraoperative blood loss, intraoperative red blood cell transfusion, intraoperative plasma transfusion, number of resected organs, number of peritoneal resection areas, number of anastomoses, intraoperative ascites, and adverse events within 30 days after surgery were recorded.

#### Parameters related to immune cells

The number of total lymphocytes B, T lymphocytes, CD4^+^T cells, CD8^+^T cells, and CD4^+^/CD8^+^T cell ratio in peripheral blood. The levels of interleukin (IL)−1β, IL-2, IL-4, IL-5, IL-6, IL-8, IL-10, IL-12p70, IL-17, tumor necrosis factor (TNF)-α, interferon (IFN)-α, and IFN-γ in peripheral blood.

#### Adverse events (AEs)

AEs were defined as complications occurring within 30 days after CRS + HIPEC. According to Sugarbaker’ textbook on peritoneal metastasis [18], AEs were divided into 5 grades: Grade I, asymptomatic and self-limited; Grade II, symptomatic and requiring medical treatment; Grade III, requiring invasive intervention; Grade IV, requiring ICU admission or reoperation; and Grade V, postoperative death. Serious adverse events (SAEs) included grade III-V AEs.

#### Survival data

The survival status, time, and cause of death of patients were recorded through outpatient follow-up or telephone interviews. The last follow-up was on February 28, 2025. The follow-up rate was 100%. Overall survival (OS) was defined as the interval from the date of diagnosis to the end of follow-up or death due to disease.

### Statistical analysis

Data analysis was performed using IBM SPSS Statistics for Windows, Version 27.0 (IBM Corp., Armonk, NY, USA). Continuous variables were expressed as median (range) or mean ± standard deviation (SD), and a t-test or rank sum test was used for comparison between groups. Categorical variables were expressed as frequencies (percentages), and the χ² test or Fisher’s exact test was used. The Kaplan-Meier method was used to calculate OS, and the Log-rank test was used for comparison between groups. The Cox proportional hazards model was used to analyze independent prognostic factors. A two-sided *P* < 0.05 was considered statistically significant. Model construction was performed in the R language (v4.3.1) using the caret and lm packages; model fitting and cross-validation codes are described in the Supplementary Material.

## Results

### Main clinicopathological features and NK cell characteristics of MPM patients

A total of 80 patients with MPM were enrolled in the preoperative study, including 35 male (43.7%) and 45 female (56.3%) patients, with a median age of 56 years (range: 14–74 years). The median preoperative BMI was 22.2 kg/m^2^ (range: 13.6–29.3 kg/m^2^), and the median KPS score was 90 (range: 80–100). Preoperative thrombosis was observed in three patients (3.7%). Histologically, 58 cases (72.5%) were classified as epithelioid type, while 22 cases (27.5%) were non-epithelioid type. Before surgery, 24 patients (30.0%) received no treatment or chemotherapy, 33 patients (41.2%) underwent targeted therapy, and 23 patients (28.8%) received immunotherapy. Surgical parameters revealed that the median duration of CRS + HIPEC was 451 min (range: 102–886 min). The median number of organs resected was 2 (range: 0–6), and the median number of peritoneal regions stripped was 6 (range: 0–8). The median PCI was 28 (range: 1–39), and a CC0-1 score was achieved in 57 patients (71.3%). Anastomosis was performed in 36 patients (45.0%), and lymph node metastasis occurred in 9 cases (11.3%). Serious adverse events within 30 days post-surgery were reported in 16 patients (20.0%), and the median length of hospital stay was 13 days (range: 6–75 days). Preoperatively, the median PB-NK cell count was 137 cells/µL (range: 16–778 cells/µL). Decreased NK cell counts were observed in 41 patients (51.3%), normal NK cell counts in 36 patients (45.0%), and increased NK cell counts in 3 patients (3.7%) (Table 1).

A total of 64 patients with MPM were enrolled in the postoperative study, with detailed clinic-pathologic information similar to the preoperative cohort. (Table 1).

**Table 1:** Clinicopathological characteristics of MPM patients in this study

Variables	Preoperative group value (*n* = 80)	Postoperative group value (*n* = 64)
Gender, n (%)		
Female	45 (56.3)	38 (59.4)
Male	35 (43.7)	26 (40.6)
Age (years), Median (range)	56 (14–74)	56 (14–74)
BMI (kg/m^2^), Median (range)	22.2 (13.6–29.3)	23.0 (13.6–29.3)
KPS, Median (range)	90 (80–100)	90 (80–100)
Preoperative thrombosis, n (%)		
No	77 (96.3)	62 (95.4)
Yes	3 (3.7)	3 (4.6)
Pathological type, n (%)		
Epithelioid type	58 (72.5)	44 (68.8)
Non-epithelioid type	22 (37.5)	20 (21.2)
Preoperative Interventions, n (%)		
No treatment or chemotherapy	24 (30.0)	47 (73.4)
Targeted therapy	33 (41.2)	9 (14.1)
Immunotherapy	23 (28.8)	8 (12.5)
Lymphatic metastasis, n (%)		
No	71 (88.7)	60 (93.8)
Yes	9 (11.3)	4 (6.2)
Procedure Time (minutes), Median (range)	451 (102–886)	445 (102–886)
Organ resections, Median (range)	2 (0–6)	2 (0–6)
Peritoneal resections, Median (range)	6 (0–9)	6 (0–9)
Anastomoses, n (%)		
No	44 (55.0)	38 (59.4)
Yes	36 (45.0)	26 (40.6)
PCI, Median (range)	28 (1–39)	28 (1–39)
CC, n (%)		
0 ~ 1	57 (71.3)	47 (73.4)
2 ~ 3	23 (28.7)	17 (26.6)
Bleeding (mL), Median (range)	100 (20–500)	100 (20–500)
RBC transfusion (U), Median (range)	0 (0–6)	0 (0–6)
Plasma transfusion (mL), Median (range)	600 (0-1200)	600 (0-1200)
Ascites volume (mL), Median (range)	200 (0-6000)	200 (0-6000)
SAEs, n (%)	16 (20.0%)	11 (17.2)
Length of stay (d), Median (range)	13 (6–75)	13 (6–31)
Preoperative NK cells (cells/µL), Median (range), n (%)	137 (16–778)	278 (22-1349)
< 155	41 (51.3)	20 (31.3)
155–550	36 (45.0)	31 (48.4)
≥ 550	3 (3.7)	13 (20.3)

*MPM* malignant peritoneal mesothelioma, *BM*I body mass index, *KPS *Karnofsky performance status score, *PCI* peritoneal cancer index, *CC* degree of tumor cell reduction score, *RBC* red blood cell*, SAEs* serious adverse events

### Comparison of main clinicopathological features between groups

The presence or absence of preoperative thrombosis was independently associated with changes in NK cells among the preoperative PB-NK cell groups (decreased, normal, and increased) (*P* = 0.023). Other factors, such as gender, age, and KPS score, did not show significant differences between the two groups (*P* > 0.05) (Table 2). The KPS score was independently correlated with changes in NK cells among the postoperative PB-NK cell groups (decreased, stable, and increased) (*P* = 0.048). Other factors, such as gender and age, were not significantly different between the two groups (*P* > 0.05) (Table 2).

**Table 2:** Major clinicopathological characteristics of MPM patients stratified NK grouping

Variables	Preoperative NK cell count			*P* value	Postoperative NK cell change			*P* value
	Decrease group (*n* = 41)	Normal group (*n* = 36)	Increase group (*n* = 3)		Decrease group (*n* = 16)	Stable group (*n* = 9)	Increase group (*n* = 39)	
Gender, n (%)				0.552				0.671
Female	25 (61.0)	19 (52.8)	1 (33.3)		11 (68.8)	5 (55.6)	22 (56.4)	
Male	16 (39.0)	17 (47.2)	2 (66.7)		5 (31.3)	4 (44.4)	17 (43.6)	
Age (years), n (%)				0.430				0.863
< 60	30 (73.2)	23 (63.9)	2 (66.7)		11 (68.8)	7 (77.8)	27 (70.3)	
≥ 60	11 (26.8)	13 (36.1)	1 (33.3)		5 (31.3)	2 (22.2)	12 (30.8)	
BMI (kg/m^2^), n (%)				0.266				0.827
< 18.5	3 (7.3)	1 (2.8)	0 (0.0)		0 (0.0)	0 (0.0)	3 (7.7)	
18.5–24.0	28 (68.3)	23 (63.9)	2 (66.7)		12 (75.0)	4 (44.4)	23 (59.0)	
≥ 24.0	10 (24.4)	12 (33.3)	1 (33.3)		4 (25.0)	5 (55.6)	13 (33.3)	
Abdominal circumference (cm), n (%)				0.243				0.621
≤ 85	27 (65.9)	22 (61.1)	3 (100.0)		10 (62.5)	4 (44.4)	24 (61.5)	
< 85	14 (34.1)	14 (38.9)	0 (0.0)		6 (37.5)	5 (55.6)	15 (38.5)	
Surgery history, n (%)				0.211				0.125
No	14 (34.1)	10 (27.8)	0 (0.0)		2 (12.5)	5 (55.6)	13 (33.3)	
Yes	27 (65.9)	25 (69.4)	3 (100.0)		14 (87.6)	4 (44.4)	26 (66.7)	
PSS, n (%)				0.415				0.584
0/1	27 (65.9)	20 (55.6)	1 (33.3)		8 (50.0)	6 (66.7)	25 (64.1)	
2/3	14 (34.1)	16 (44.4)	2 (66.7)		8 (50.0)	3 (33.3)	14 (35.9)	
Preoperative IPc, n (%)				0.762				0.549
No	38 (92.7)	35 (97.2)	2 (66.7)		15 (93.8)	9 (100.0)	38 (96.9)	
Yes	3 (7.3)	1 (2.8)	1 (33.3)		1 (6.3)	0 (0.0)	1 (2.6)	
KPS, n (%)				0.099				**0.048**
< 90	22 (53.7)	20 (55.6)	0 (0.0)		7 (43.8)	2 (22.2)	25 (64.1)	
≥ 90	19 (46.3)	16 (44.4)	3 (100.0)		9 (56.3)	7 (77.8)	14 (35.9)	
Thrombosis history, n (%)				0.190				0.549
No	39 (95.1)	36 (100.0)	3 (100.0)		15 (93.8)	9 (100.0)	38 (96.9)	
Yes	2 (4.9)	0 (0.0)	0 (0.0)		1 (6.3)	0 (0.0)	1 (2.6)	
Preoperative thrombosis, n (%)				**0.023**				0.527
No	40 (97.6)	35 (97.2)	2 (66.7)		16 (100.0)	8 (88.9)	37 (94.9)	
Yes	1 (2.4)	1 (2.8)	1 (33.3)		0 (0.0)	1 (11.1)	2 (5.1)	
Pathological types, n (%)				0.408				0.556
Epithelioid	31 (75.6)	22 (61.1)	3 (100.0)		13 (81.3)	3 (33.3)	28 (71.8)	
Non-epithelioid	10 (24.4)	14 (38.9)	0 (0.0)		3 (18.8)	6 (66.7)	11 (28.2)	
Vascular tumor emboli, n (%)				0.190				0.527
No	37 (90.2)	35 (97.2)	3 (100.0)		16 (100.0)	8 (88.9)	37 (94.9)	
Yes	4 (9.8)	1 (2.8)	0 (0.0)		0 (0.0)	1 (11.1)	2 (5.1)	
Lymphatic metastasis, n (%)				0.654				0.124
No	36 (87.8)	32 (88.9)	3 (100.0)		16 (100.0)	9 (100.0)	35 (89.7)	
Yes	5 (12.2)	4 (11.1)	0 (0.0)		0 (0.0)	0 (0.0)	4 (10.3)	
Ki-67 index, n (%)				0.920				0.671
≤ 9%	13 (31.7)	13 (36.1)	1 (33.3)		5 (31.3)	4 (44.4)	17 (43.6)	
> 9%	28 (68.3)	23 (63.9)	2 (66.7)		11 (68.8)	5 (55.6)	22 (56.4)	
Therapeutic Interventions, n (%)				0.698				0.815
No treatment or chemotherapy	12 (29.3)	12 (33.3)	0 (0.0)		12 (75.0)	6 (66.7)	29 (74.4)	
Targeted therapy	18 (43.9)	13 (36.1)	2 (66.7)		3 (18.8)	1 (11.1)	5 (12.8)	
Immunotherapy	11 (26.8)	11 (30.6)	1 (33.3)		1 (6.3)	2 (22.2)	5 (12.8)	

*MPM* malignant peritoneal mesothelioma, *BMI* body mass index, *PSS* score of previous surgery, *IPc* intraperitoneal chemotherapy, *KPS* karnofsky performance scale

### Comparison of CRS + HIPEC-related parameters between groups

Plasma transfusion volume (*P* = 0.004) and length of hospital stay (*P* = 0.023) were independently associated with changes in preoperative PB-NK cells. Other CRS + HIPEC-related parameters, including procedure duration, PCI, CC, and number of organ resections, did not show statistically significant differences among the three groups (*P* > 0.05) (Table 3). No independent correlation was observed between CRS + HIPEC-related parameters and changes in PB-NK cells after surgery (*P* > 0.05) (Table 3).

**Table 3:** Major CRS + HIPEC related characteristics of MPM patients stratified by NK grouping

Variables	Preoperative NK cell count			*P* value	Postoperative NK cell change			*P* value
	Decrease group (*n* = 41)	Normal group (*n* = 36)	Increase group (*n* = 3)		Decrease group (*n* = 16)	Stable group (*n* = 9)	Increase group (*n* = 39)	
Procedure Time (minutes), n (%)				0.469				0.985
≤ 451	19 (46.3)	21 (58.3)	1 (33.3)		9 (56.3)	5 (55.6)	21 (53.8)	
> 451	22 (53.7)	15 (41.7)	2 (66.7)		7 (43.8)	4 (44.4)	18 (46.2)	
PCI, n (%)				0.737				0.474
≤ 28	20 (48.8)	20 (55.6)	2 (66.7)		10 (62.5)	6 (66.7)	19 (48.7)	
> 28	21 (51.2)	16 (44.4)	1 (33.3)		6 (37.5)	3 (33.3)	20 (51.3)	
CC, n (%)				0.643				0.615
0 ~ 1	28 (68.3)	27 (75.0)	2 (66.7)		13 (81.3)	7 (77.8)	27 (69.2)	
2 ~ 3	13 (31.7)	9 (25.0)	1 (33.3)		3 (18.8)	2 (22.2)	12 (30.8)	
Bleeding (mL), n (%)				0.191				0.815
≤ 100	18 (43.9)	23 (63.9)	2 (66.7)		10 (62.5)	6 (66.7)	22 (56.4)	
> 100	23 (56.1)	13 (36.1)	1 (33.3)		6 (37.5)	3 (33.3)	17 (43.6)	
RBC transfusion, n (%)				0.356				0.714
No	22 (53.7)	25 (69.4)	2 (66.7)		11 (68.8)	7 (77.8)	25 (64.1)	
Yes	19 (46.3)	11 (30.6)	1 (33.3)		5 (31.3)	2 (22.2)	14 (35.9)	
Plasma transfusion (mL), n (%)				**0.004**				0.610
≤ 600	19 (46.3)	29 (80.6)	1 (33.3)		11 (68.8)	7 (77.8)	24 (61.5)	
> 600	22 (53.7)	7 (19.4)	2 (66.7)		5 (31.3)	2 (22.2)	15 (38.5)	
Organ resections, n (%)				0.215				0.098
≤ 2	25 (61.0)	21 (58.3)	3 (100.0)		8 (50.0)	4 (44.4)	29 (74.4)	
> 2	16 (39.0)	15 (41.7)	0 (0.0)		8 (50.0)	5 (55.6)	10 (25.6)	
Peritoneal resections, n (%)				0.884				0.188
≤ 6	25 (61.0)	21 (58.3)	2 (66.7)		10 (62.5)	8 (88.9)	23 (59.0)	
> 6	16 (39.0)	15 (41.7)	1 (33.3)		6 (37.5)	1 (11.1)	16 (41.0)	
Anastomose, n (%)				0.137				0.134
No	23 (56.1)	18 (50.0)	3 (100.0)		7 (43.8)	5 (55.6)	27 (69.2)	
Yes	18 (43.9)	18 (50.0)	0 (0.0)		9 (56.3)	4 (44.4)	12 (30.8)	
Ascites volume (mL), n (%)				0.394				0.388
0	13 (31.7)	11 (30.6)	3 (100.0)		7 (43.8)	3 (33.3)	12 (30.8)	
0 ~ 1000	18 (43.9)	15 (41.7)	0 (0.0)		4 (25.0)	5 (55.6)	20 (51.3)	
> 1000	10 (24.4)	10 (27.8)	0 (0.0)		5 (31.3)	1 (11.1)	7 (17.9)	
Length of stay (d), n (%)				**0.023**				0.267
≤ 13	19 (46.3)	25 (69.4)	2 (66.7)		8 (50.0)	5 (55.6)	28 (71.8)	
> 13	22 (53.7)	11 (30.6)	1 (33.3)		8 (50.0)	4 (44.4)	11 (28.2)	
SAEs, n (%)				0.097				0.514
No	30 (73.2)	31 (86.1)	3 (100.0)		12 (75.0)	7 (77.8)	34 (87.2)	
Yes	11 (26.8)	5 (13.9)	0 (0.0)		4 (25.0)	2 (22.2)	5 (12.8)	

*MPM* malignant peritoneal mesothelioma, *PCI *peritoneal cancer index, *CC* degree of tumor cell reduction score, *SAEs* serious adverse events

### Comparison of immune cell-related parameters between groups

The immune cell-related parameters independently correlated with the changes in PB-NK cells before surgery included the total lymphocyte count (*P* = 0.011) and the CD4^+^/CD8^+^ T lymphocyte ratio (*P* = 0.018). Other immune cell-related parameters, such as B lymphocytes and IL-2, did not show statistically significant differences between the two groups (*P* > 0.05) (Table 4).

Post-surgery, the immune cell-related parameters independently associated with changes in peripheral blood NK cells included IL-4 (*P* = 0.020), IL-5 (*P* = 0.007), IL-6 (*P* = 0.016), and IL-8 (*P* = 0.018). Other immune cell-related parameters, such as B lymphocytes and IL-1β, did not exhibit statistically significant differences between the two groups (*P* > 0.05) (Table 4).

**Table 4:** Major immune-related characteristics of MPM patients stratified by NK grouping

Variables	Preoperative NK cell count			*P* value	Postoperative NK cell change			*P* value
	Decrease group (*n* = 41)	Normal group (*n* = 36)	Increase group (*n* = 3)		Decrease group (*n* = 16)	Stable group (*n* = 9)	Increase group (*n* = 39)	
lymphocyte (cells/µL), n (%)				**0.011**				0.090
< 1100	14 (34.1)	4 (11.1)	0 (0.0)		4 (25.0)	4 (44.4)	5 (12.8)	
≥ 1100	27 (65.9)	32 (88.9)	3 (100.0)		12 (75.0)	5 (55.6)	34 (87.2)	
B lymphocyte (cells/µL), n (%)				0.567				0.869
< 90	17 (41.5)	13 (36.1)	2 (66.7)		8 (50.0)	4 (44.4)	21 (53.8)	
≥ 90	24 (58.5)	23 (63.9)	1 (33.3)		8 (50.0)	5 (55.6)	18 (46.2)	
T lymphocyte (cells/µL), n (%)				0.335				0.692
< 940	18 (43.9)	10 (27.8)	1 (33.3)		7 (43.8)	4 (44.4)	13 (33.3)	
≥ 940	23 (56.1)	26 (72.2)	2 (66.7)		9 (56.3)	5 (55.6)	26 (66.7)	
CD4^+^T lymphocyte (cells/µL), n (%)				0.495				0.547
< 410	10 (24.4)	5 (13.9)	1 (33.3)		5 (31.3)	4 (44.4)	10 (25.6)	
≥ 410	31 (75.6)	31 (86.1)	2 (66.7)		11 (68.8)	5 (55.6)	29 (74.4)	
CD8^+^T lymphocyte (cells/µL), n (%)				0.242				0.242
< 240	10 (24.4)	6 (16.7)	0 (0.0)		3 (18.8)	1 (11.1)	3 (7.7)	
≥ 240	31 (75.6)	30 (83.3)	3 (100.0)		13 (81.3)	8 (88.9)	36 (92.3)	
CD4^+^/CD8^+^T lymphocyte, n (%)				**0.018**				0.882
< 0.9	6 (14.6)	3 (8.3)	2 (66.7)		4 (25.0)	3 (33.3)	6 (18.2)	
≥ 0.9	35 (85.4)	33 (91.7)	1 (33.3)		12 (75.0)	6 (66.7)	27 (81.8)	
IL-1β (pg/mL), n (%)				*				*
≤ 24.13	41 (100.0)	36 (100.0)	3 (100.0)		16 (100.0)	9 (100.0)	39 (100.0)	
> 24.13	0 (0.0)	0 (0.0)	0 (0.0)		0 (0.0)	0 (0.0)	0 (0.0)	
IL-2 (pg/mL), n (%)				0.883				0.052
≤ 4.96	36 (87.8)	31 (86.1)	3 (100.0)		12 (75.0)	9 (100.0)	38 (97.4)	
> 4.96	5 (12.2)	5 (13.9)	0 (0.0)		4 (25.0)	0 (0.0)	1 (2.6)	
IL-4 (pg/mL), n (%)				0.284				**0.020**
≤ 3.54	32 (78.0)	24 (66.7)	2 (66.7)		16 (100.0)	8 (88.9)	24 (72.7)	
> 3.54	9 (22.0)	12 (33.3)	1 (33.3)		0 (0.0)	1 (11.1)	9 (27.3)	
IL-5 (pg/mL), n (%)				*				**0.007**
≤ 7.12	41 (100.0)	36 (100.0)	3 (100.0)		12 (75.0)	9 (100.0)	39 (100.0)	
> 7.12	0 (0.0)	0 (0.0)	0 (0.0)		4 (25.0)	0 (0.0)	0 (0.0)	
IL-6 (pg/mL), n (%)				0.787				**0.016**
≤ 15.02	27 (65.9)	25 (69.4)	2 (66.7)		6 (37.5)	5 (55.6)	33 (84.6)	
> 15.02	14 (34.1)	11 (30.6)	1 (33.3)		10 (62.5)	4 (44.4)	6 (15.4)	
IL-8 (pg/mL), n (%)				0.614				**0.018**
≤ 53.09	38 (92.7)	34 (94.4)	3 (100.0)		13 (81.3)	6 (66.7)	39 (100.0)	
> 53.09	3 (7.3)	2 (5.6)	0 (0.0)		3 (18.8)	3 (33.3)	0 (0.0)	
IL-10 (pg/mL), n (%)				0.210				0.288
≤ 6.23	24 (58.5)	23 (63.9)	3 (100.0)		12 (75.0)	5 (55.6)	29 (74.4)	
> 6.23	17 (41.5)	13 (36.1)	0 (0.0)		4 (25.0)	4 (44.4)	7 (25.6)	
IL-12p70 (pg/mL), n (%)				0.929				0.168
≤ 5.32	39 (95.1)	34 (94.4)	3 (100.0)		16 (100.0)	8 (88.9)	36 (92.3)	
> 5.32	2 (4.9)	2 (5.6)	0 (0.0)		0 (0.0)	1 (11.1)	3 (7.7)	
IL-17 (pg/mL), n (%)				0.357				0.434
≤ 28.25	40 (97.6)	36 (100.0)	3 (100.0)		16 (100.0)	8 (88.9)	38 (97.4)	
> 28.25	1 (2.4)	0 (0.0)	0 (0.0)		0 (0.0)	1 (11.1)	1 (2.6)	
TNF-α (pg/mL), n (%)				0.357				0.434
≤ 17.11	40 (97.6)	36 (100.0)	3 (100.0)		16 (100.0)	8 (88.9)	38 (97.4)	
> 17.11	1 (2.4)	0 (0.0)	0 (0.0)		0 (0.0)	1 (11.1)	1 (2.6)	
IFN-α (pg/mL), n (%)				0.190				0.434
≤ 12.57	39 (95.1)	36 (100.0)	3 (100.0)		16 (100.0)	8 (88.9)	38 (97.4)	
> 12.57	2 (4.9)	0 (0.0)	0 (0.0)		0 (0.0)	1 (11.1)	1 (2.6)	
IFN-γ (pg/mL), n (%)				0.967				0.350
≤ 3.13	25 (61.0)	22 (61.1)	2 (66.7)		15 (93.8)	6 (66.7)	29 (74.4)	
> 3.13	16 (39.0)	14 (38.9)	1 (33.3)		1 (6.3)	3 (33.3)	7 (25.6)	

*MPM* malignant peritoneal mesothelioma,* IL* interleukin, *TNF* tumor necrosis factor, *IFN* interferon-gamma

*: this variable was constant, and no calculation was performed

### Multivariate analysis

In the multivariate logistic regression analysis, factors with *P* < 0.05 in the univariate analysis were included. The results indicated that plasma infusion volume (*P* = 0.013) was an independent correlated factor for preoperative NK cell reduction (Table 5). Furthermore, the patient’s overall clinical status, as indicated by KPS score (*P* = 0.048), was determined to be an independent associated factor for postoperative NK cell exhaustion (Table 5).

**Table 5:** Multivariate analysis of MPM patients in preoperative/postoperative NK cell count group

Variables	Wald	OR	95% CI	*P* value
Preoperative PB-NK cell group				
Plasma transfusion (> 600 mL vs. ≤ 600 mL)	6.176	0.242	[0.079, 0.741]	**0.013**
Preoperative PB-NK cell group				
KPS (≥ 90 vs. < 90)	3.898	0.166	[0.028, 0.987]	**0.048**

*MPM* malignant peritoneal mesothelioma, *KPS* karnofsky performance scale

### Survival analysis

**Fig. 1:**
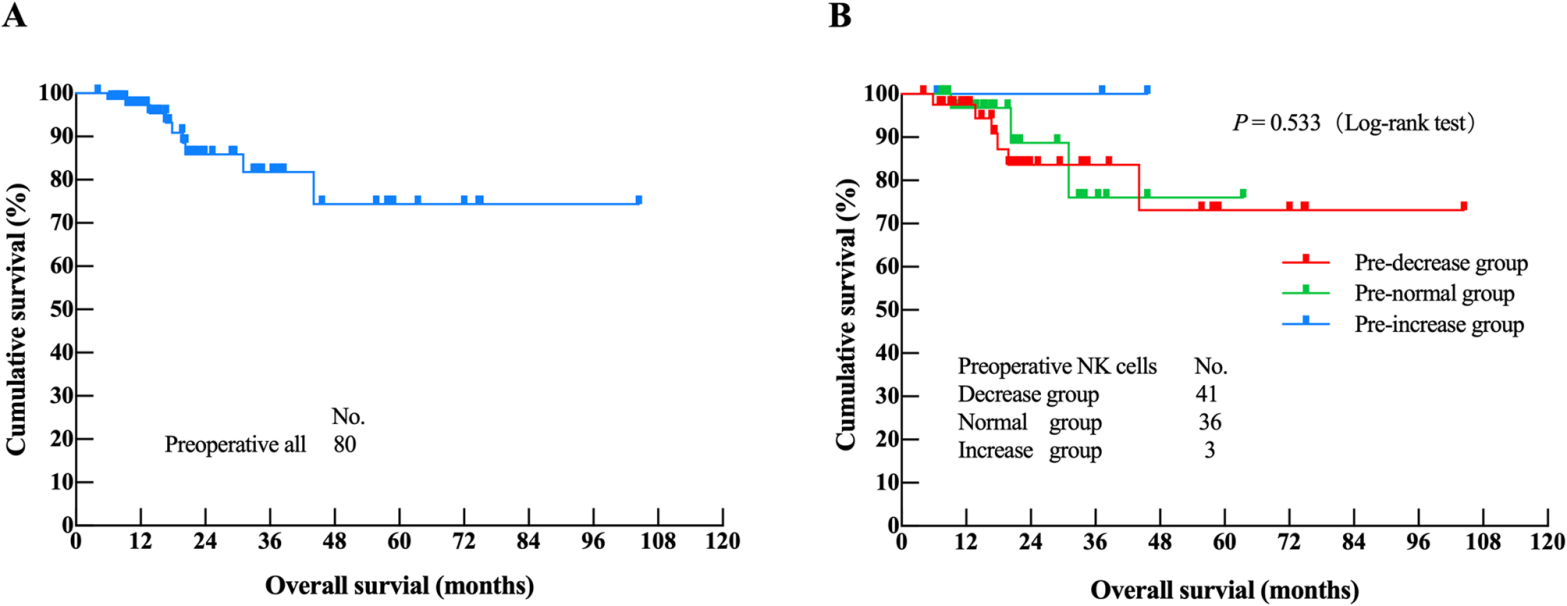
Preoperative survival analysis: (**A**) Overall Survival analysis of 80 MPM patients before surgery; (**B**) NK cell decreased group, normal group and increased group

As of February 28, 2025, the median OS of 80 patients with MPM had not been reached. Among these patients, 9 (11.3%) had died, and 71 (88.7%) remained alive. No significant difference was observed in OS among the three preoperative PB-NK cell groups (decreased, normal, and increased) (*P =* 0.533). Kaplan-Meier analysis of the 80 patients revealed that clinicopathological factors independently associated with MPM prognosis included PSS, abdominal circumference, PCI score, intraoperative blood loss, ascites, vascular tumor thrombus, lymph node metastasis, preoperative CD8^+^ T lymphocyte count, and IL-17 value (*P* < 0.05) (Supplementary Table S1). Factors with *P* < 0.05 in univariate survival analysis were included in Cox multivariate analysis which identified 4 independent prognostic factors associated with prognosis: the PSS score (*P* = 0.015), lymph node metastasis (*P* = 0.015), intraoperative blood loss (*P* = 0.013), and CD8^+^ T lymphocytes (*P* = 0.001) (Table 6). Higher preoperative NK levels showed a non-significant trend toward improved OS (HR = 0.674, *P* = 0.533) (Fig. 1).

**Fig. 2:**
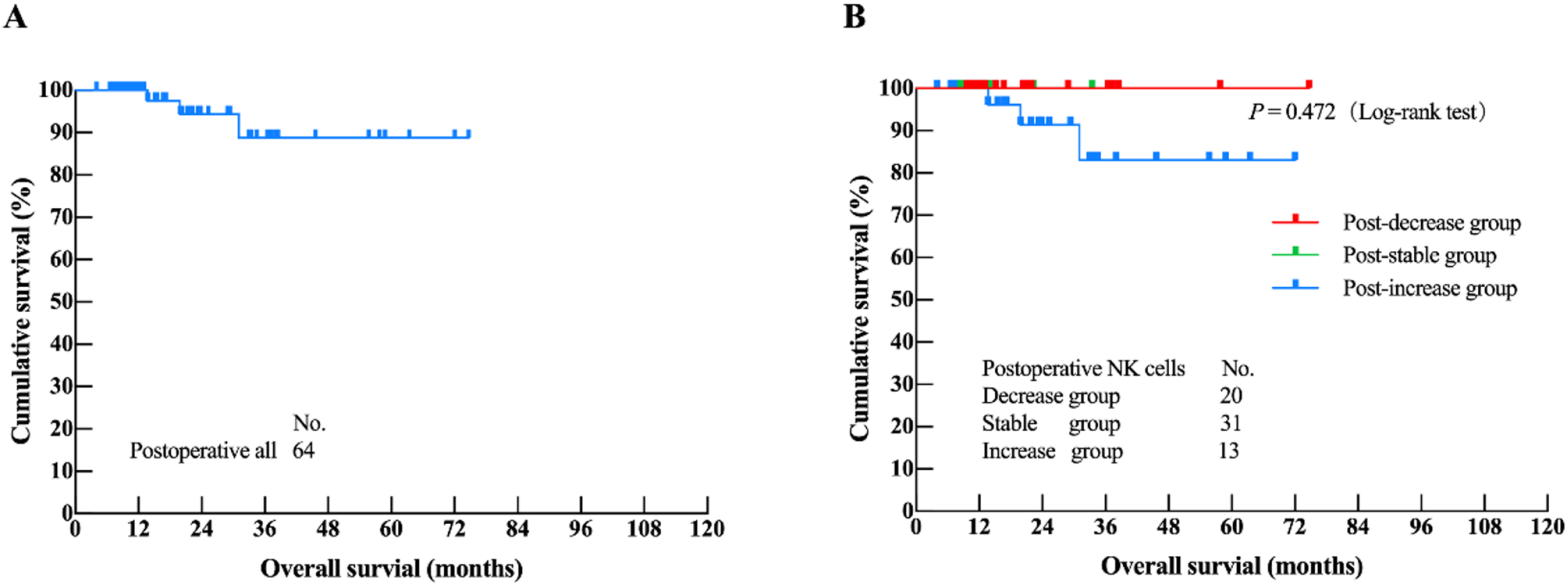
Postoperative survival analysis: (**A**) Overall Survival analysis of 64 MPM patients after surgery; (**B**) NK cells decreased, stable and increased group

As of February 28, 2025, the median OS of 64 MPM patients had not been reached. Among these patients, 3 (4.7%) had deceased and 61 (95.3%) remained alive. No significant difference was observed in OS among the groups with decreased, stable, and increased postoperative PB-NK cells (*P =* 0.472). KM analysis of the 64 patients revealed that the clinicopathological factors independently associated with MPM prognosis were postoperative IL-17, TNF-α, and IFN-α levels (*P* < 0.05) (Supplementary Table S1). Upon incorporation into Cox regression analysis, the results indicated that the clinicopathological factor independently correlated with MPM prognosis was the postoperative IL-17 value (*P* = 0.013) (Table 6). Notably, the change in NK cell counts did not exhibit a clear association with OS (*P* = 0.472, HR = 7.728) (Fig. 2).

**Table 6:** Analysis of survival factors of MPM patients in this study

Variables	Wald	HR	95% CI	*P* value
Preoperative PB-NK cell group				
PSS (0/1 vs. 2/3)	5.948	0.054	[0.005, 0.564]	**0.015**
Lymphatic metastasis (Yes vs. No)	5.902	8.266	[1.504, 45.432]	**0.015**
Bleeding (> 100 mL vs. ≤ 100 mL)	6.130	0.086	[0.012, 0.600]	**0.013**
CD8^+^T lymphocyte (≥ 240 cells/µL vs. < 240 cells/µL)	11.397	0.018	[0.002, 0.186]	**0.001**
Postoperative PB-NK cell group				
Postoperative IL-17 (> 28.25pg/mL vs. ≤ 28.25pg/mL)	6.212	0.029	[0.002, 0.470]	**0.013**

*MPM* malignant peritoneal mesothelioma, *PSS* score of previous surgery, *IL* interleukin

### Prediction model for NK cell change

#### Statistical analysis

The levels of NK cells and immune-related parameters were measured in 64 patients at various time points post-surgery. The change in PB-NK cell levels (NK_Change) was defined as the difference between the current measurement and the previous measurement, with the timepoint spanning from the last examination to the current one (in months). A total of 120 groups of NK cell change/time data were collected. Univariate analysis identified variables significantly associated with NK cell changes (*P* < 0.2), among which the statistically significant variables were as follows: preoperative NK cell count (*P* = 1.77 × 10^−9^), postoperative NK cell count (*P* = 4.22 × 10^−4^), postoperative total lymphocyte count (*P* = 5.35 × 10^−4^), postoperative T cell count (*P* = 0.037), and postoperative CD8^+^T cell count (*P* = 0.026). To account for heteroscedasticity, the weighted least squares (WLS) method was employed, using the inverse square of the fitted values from the ordinary least squares (OLS) model as weights. The final predictive model incorporated the following predictors: postoperative NK cell level (Post_NK), preoperative NK cell level (Pre_NK), centered peritoneal cancer index (PCI_centered, calculated as PCI - mean (PCI)), postoperative CD8^+^T cell count (Post_CD8), and follow-up time (Timepoint).

#### Model specification

The regression equation is expressed as:

NK_Change_*i*_ = *β*_0_ + *β*_1_Post_NK_*i*_ + *β*_2_Pre_NK_*i*_ + *β*_3_PCI_centered_*i*_ + *β*_4_Post_CD8_*i*_ + *β*_5_Timepoint_*i*_+*ϵ*_*i*_, the weight *w*_*i*_=1/$\hat{y}$_i_^2^ is derived from the fitted value $\hat{y}$_i_^2^ for the OLS model.

#### Regression coefficients and model performance

The developed model demonstrated robust explanatory power and universal applicability. All regression coefficients and statistical metrics exhibited significant statistical significance: the adjusted R² was 0.810 (indicating that 81.0% of the variance was explained); the residual standard error (RSE) was 1.440, representing a substantial improvement compared to the original model (RSE = 252.8); the F-statistic was 94.01 (*P* < 0.001), confirming the global significance of the model. The root mean square error (RMSE) of cross-validation was 88.755, reflecting stable performance on unseen data (Table 7).

#### Model interpretation

The model revealed that an increase of one unit in the Post_NK level was associated with an increase of 0.386 units in NK_Change (*P* < 0.001). A higher baseline NK level (Pre_NK) was independently associated with a decrease in NK cell change (NK_Change) (β = −0.391, *P* < 0.001), reflecting both mathematical constraint and biological baseline effects. An increase in PCI_centered was independently associated with an increase in NK cell changes (β = −1.143, *P* < 0.001). For each additional follow-up month (Timepoint), NK_Change increased by 8.068 units (*P* < 0.001). The level of CD8 + T cells (Post_CD8) exhibited a synergistic effect with the change in NK cells (NK_Change) (β = 0.050, *P* < 0.001).

Significant collinearity was detected between the categorical variable of treatment (Treatment_Group) and the time variable (Timepoint) in the dataset using the generalized variance inflation factor (GVIF) and Spearman’s rank correlation test.

The GVIF^1/(2Df)^ values were as follows:

Treatment_Group, 3.76 (GVIF = 199.05, Df = 2).

Treatment_Group × Timepoint interaction term, 3.99 (GVIF = 253.18, Df = 2). 

Both values exceeded the threshold of 2, suggesting a potential risk of multicollinearity.

Spearman rank correlation coefficient: there is a strong correlation between Treatment_Group and Timepoint (ρ = 0.82, *P* < 0.001), indicating high synchronization between these two variables. Consequently, during model construction, the Treatment_Group variable was excluded, and only the Timepoint variable was retained to ensure model stability and interpretability.

**Table 7:** Regression coefficients and statistical significance of the model analysis of the amount of MPM patients NK cell change

Variables	Estimated (β)	Std. error	T value	95% CI	*P* value
(Intercept)	−59.137	0.414	−9.220	[−71.900, −46.374]	**< 0.001**
Post_NK	0.386	0.053	7.330	[0.282, 0.491]	**< 0.001**
Pre_NK	−0.391	0.065	−5.978	[−0.521, −0.261]	**< 0.001**
PCI_centered	−1.143	0.278	−4.107	[−1.690, −0.591]	**< 0.001**
Post_CD8^+^T	0.050	0.012	4.117	[0.026, 0.074]	**< 0.001**
Timepoint	8.068	1.303	6.193	[5.480, 10.656]	**< 0.001**

*MPM* malignant peritoneal mesothelioma; *Std.* standard; *CI* confidence interval; Post_NK: preoperative peripheral blood NK cell count; Pre_NK: postoperative peripheral blood NK cell count; *PCI *peritoneal carcinomatosis index; PCI_centered: equal to the PCI raw value minus the mean PCI for all patients in the data; Post_CD8^+^T: postoperative peripheral blood CD8^+^T cell count; Timepoint: time span of the patient from the last examination to the present examination.

## Discussion

This study of 80 preoperative and 64 postoperative MPM patients revealed a 51.3% preoperative PB-NK depletion rate (< 150 cells/µL), declining to 31.3% postoperatively. Median PB-NK cells increased 2.03-fold (137 to 278 cells/µL), with 60.9% of patients showing postoperative NK elevation. Preoperative NK depletion correlated with thrombosis risk (*P* = 0.023), intraoperative plasma transfusion (*P* = 0.004), and hospitalization duration (*P* = 0.023). Postoperatively, NK dynamics associated with KPS (*P* = 0.048) and elevated IL-4/5/6/8 (*P* < 0.05). Multivariate analysis confirmed plasma infusion volume as an independent associated factor for preoperative NK reduction (*P* = 0.013), while low KPS predicted postoperative NK decline (*P* = 0.048).

Survival analysis identified poor prognostic factors: high PSS (*P* = 0.015), lymph node metastasis (*P* = 0.015), substantial intraoperative blood loss (*P* = 0.013), low preoperative CD8⁺ T cells (*P* = 0.001), and low postoperative IL-17 (*P* = 0.013). While preoperative NK elevation did not reach statistical significance for OS, its biological relevance merits further study. Multivariate regression confirmed significant influences on NK dynamics: baseline NK (*P* < 0.001), postoperative NK (*P* < 0.001), PCI (*P* < 0.001), CD8⁺ T cells (*P* < 0.001), and recovery duration (*P* < 0.001).

Preoperative PB-NK depletion correlated with thrombosis risk and intraoperative plasma requirements, reflecting tumor immune escape. High tumor burden suppresses NK progenitors via TGF-β [14], increasing surgical risks. NK cells may maintain microenvironment homeostasis through IFN-γ-mediated tumor control [19] though direct coagulation links remain unproven.

In this cohort, the observed increase in NK cells following CRS + HIPEC suggests a potential immunomodulatory effect. However, confounding factors cannot be excluded, such as the presence of postoperative complications or persistent inflammation among these patients. Although this observation is consistent with reports of NK cell recovery after tumor resection in other malignancies [20], there is currently no direct evidence establishing a mechanistic link between CRS + HIPEC and NK cell reconstitution in MPM.

Using stricter multivariate regression criteria, we identified poor preoperative performance status (low KPS) as an independent factor affecting postoperative NK cell decline. This highlights the importance of optimizing patients’ condition before surgery to reduce immune system damage. Additionally, univariate analysis showed that postoperative increases in specific cytokines (e.g., IL-4/5/6/8) were negatively linked to NK cell recovery. Although studies suggest that IL-4 may promote myeloid-derived suppressor cell (MDSC) activity, which could suppress NK cells [21], and IL-5 inhibition may help preserve NK function [22], the direct causal relationship between these cytokine increases, and postoperative NK cell reduction remains unclear and requires further mechanistic research.

Multivariate Cox regression confirmed intraoperative blood loss and lymph node metastasis as poor prognostic factors, likely via TGF-β/IL-10 immunosuppression [19]. Although preoperative NK levels showed no OS benefit, dynamic NK changes positively correlated with CD8⁺ T cells (*P* < 0.001), indicating innate-adaptive synergy. High baseline CD8⁺ T cells prolonged OS, while IL-17 enhanced antitumor responses via CXCL9/10 [23].

Multivariate regression linked diminished NK recovery to high baseline NK levels and PCI (*P* < 0.001), likely reflecting TGF-β–mediated immunosuppression in high-tumor-burden settings [24, 25]. We further hypothesize an “immune ceiling effect” where maximal baseline immune activity limits postoperative recovery potential—a concept meriting future investigation. The synergistic interaction between NK cells and CD8^+^ T cells appear to be associated with the dynamic equilibrium of IFN-γ signaling. Dubrot et al. demonstrated that IFN-γ enhances antigen presentation to CD8^+^ T cells by upregulating MHC-I molecules while simultaneously inhibiting NK cell activation [26]. Single-cell sequencing is required to elucidate the spatiotemporal-specific interaction network.

Additionally, PB-NK cell counts progressively increased over time following surgery, underscoring the significance of standardized postoperative management, including antineoplastic therapy integrated with nutritional support, for promoting immune recovery. Moreover, the collinearity between treatment stage and time variables might stem from the consistency of the staged comprehensive treatment protocol for MPM patients post-surgery: within 1-month post-surgery, 92% of patients received only nutritional Support; from 1 to 6 months post-surgery, 78% of patients underwent drug sensitivity-guided chemotherapy (e.g., capecitabine/oxaliplatin) combined with targeted therapy (apatinib); from 6 to 12 months post-surgery, 65% of patients received immunotherapy (PD-1/PD-L1 inhibitors) in conjunction with targeted therapy (apatinib).

## Limitations and prospects

This study is a single-center retrospective analysis with a limited sample size (*n* = 64 for longitudinal analysis) and has several limitations. First, although the safety analysis found no significant link between NK cell kinetics and outcomes, unmeasured factors like subclinical inflammation may still affect results. Second, key NK cell functions, such as degranulation capacity, and other immune microenvironment components, including the Treg/MDSC ratio, were not assessed. Third, the multivariate analyses were exploratory and may yield false-positive results due to no correction for multiple comparisons. Lastly, baseline variables like Pre_NK could affect the interpretation of observed associations.

Future multicenter studies should combine spatial transcriptomics and scRNA-seq to (1) explore NK cell subset heterogeneity; (2) map interactions between NK cells and CD8^+^ T cells; and (3) identify key factors causing NK cell dysfunction after CRS + HIPEC.

## Conclusion

This study elucidated the dynamic changes in PB-NK cell levels among MPM patients and their clinical significance, demonstrated that CRS + HIPEC enhanced NK cell function through immune microenvironment remodeling, and established a predictive model for postoperative NK recovery. Moving forward, it is essential to integrate multi-omics approaches with prospective cohort studies to thoroughly investigate the heterogeneity of NK cell subsets and their spatiotemporal interaction networks, thereby providing precise regulatory targets for personalized immunotherapy.

## Supplementary Information

Supplementary Material 1

Supplementary Material 2

## Data Availability

No datasets were generated or analysed during the current study.
