# Supplementary material for: Dynamic evolution of NK cells and immune remodeling mediated by CRS + HIPEC: prognostic mechanisms and therapeutic implications for malignant peritoneal mesothelioma
Source: World J Surg Oncol. 2025 Nov 3;23:416. doi: 10.1186/s12957-025-04019-2 (PMC12581496; doi:10.1186/s12957-025-04019-2)
Supplement: Supplementary file 1 — Supplementary Material 1 [file 12957_2025_4019_MOESM1_ESM.docx]

# Final WLS model

lm_wls <- lm(

NK_Change_Cont ~ Post_NK + Pre_NK + PCI_centered + Post_CD8 + Timepoint,

data = final_data_clean,

weights = weights # weights = 1 / fitted(lm_ols)^2

)

# 10-fold cross-validation

train_control <- trainControl(method = "cv", number = 10)

model_cv <- train(

NK_Change_Cont ~ Post_NK + Pre_NK + PCI_centered + Post_CD8 + Timepoint,

data = final_data_clean,

method = "lm",

trControl = train_control,

weights = weights

)
