# Supplementary material for: Dynamic evolution of NK cells and immune remodeling mediated by CRS + HIPEC: prognostic mechanisms and therapeutic implications for malignant peritoneal mesothelioma
Source: World J Surg Oncol. 2025 Nov 3;23:416. doi: 10.1186/s12957-025-04019-2 (PMC12581496; doi:10.1186/s12957-025-04019-2)
Supplement: Supplementary file 2 — Supplementary Material 2 [file 12957_2025_4019_MOESM2_ESM.docx]

| **Table S1. Univariate survival analysis of MPM patients in this study.** | |
| --- | --- |
| Variables | *P* value |
| Preoperative PB-NK cell group | |
| PSS (0/1 *vs.* 2/3) | **0.015** |
| Abdominal circumference (> 85cm *vs.* ≤ 85cm) | **0.043** |
| PCI (> 28 *vs.* ≤ 28) | **0.019** |
| Bleeding (> 100mL *vs.* ≤ 100mL) | **0.029** |
| Ascites volume (0 mL *vs.* 0-1000mL *vs.*>1000mL) | **0.036** |
| Vascular tumor emboli (Yes *vs.* No) | **0.035** |
| Lymphatic metastasis (Yes *vs.* No) | **0.001** |
| CD8^+^T lymphocyte (≥ 240 cells/μL *vs.* < 240 cells/μL) | **< 0.001** |
| Preoperative IL-17 (> 28.25pg/mL *vs.* ≤ 28.25pg/mL) | **0.010** |
| Preoperative PB-NK cell group | |
| Postoperative IL-17 (> 28.25pg/mL *vs.* ≤ 28.25pg/mL) | **< 0.001** |
| Postoperative TNF-α (> 17.11pg/mL *vs.* ≤ 17.11pg/mL) | **< 0.001** |
| Postoperative IFN-α (> 12.57pg/mL *vs.* ≤ 12.57pg/mL) | **< 0.001** |

MPM: malignant peritoneal mesothelioma; PSS: score of previous surgery; PCI ：peritoneal cancer index; IL: interleukin; TNF: tumor necrosis factor; IFN: interferon-gamma.
